# Pyrogen

**Published:** 1897-03

**Authors:** W. A. Yingling

**Affiliations:** Emporia, Kansas


### PYROGEN.

EMPORIA, KANSAS, February 10th, 1897.

EDITOR OF THE HOMŒOPATHIC PHYSICIAN:

I think it would be a great benefit to the profession to have a collection of the verified and clinical symptoms of *Pyrogen*, and if you will ask your readers to send me all the symptoms *promptly cured* by the single remedy, I will arrange them for the printer in due form. I have found it the leading remedy for the grippe, and have some new verifications and symptoms, "Sensation of a cap on the head," "Sensation as if the heart were pumping cold water," are two symptoms that I have verified in my own practice, the latter a new indication for the remedy.

Fraternally,
W. A. YINGLING.

